# Crocin Bleaching Assay Using Purified Di-gentiobiosyl Crocin (-crocin) from Iranian Saffron

**Published:** 2011

**Authors:** Seyedeh Zahra Bathaie, Azam Shams, Fatemeh Moghadas Zadeh Kermani

**Affiliations:** 1 *Department of Clinical Biochemistry, Faculty of Medical Sciences, Tarbiat Modares University, Tehran, Iran*; 2 *Department of Biochemistry, Payame Noor University, Tehran, Iran*; 3 *Children Medical Center, Tehran University of Medical Sciences, Tehran, Iran*

**Keywords:** Antioxidants, -Crocin, Carotenoids, Crocin Bleaching Assay, Iranian Saffron

## Abstract

**Objective(s):**

Crocin bleaching assay (CBA) is a new method for determination of antioxidant capacity. In CBA, addition of hydrogen to the conjugated double bonds of crocin results in reduction of crocin and increase in the absorbance at 440 nm, which is considered as a measure of antioxidant potential. Here CBA method was set up using di-gentiobiosyl crocin or α-crocin from Iranian saffron. Then, the antioxidant activity of some known antioxidants i.e. L-ascorbic acid, bilirubin, Trolox, uric acid, and some plasma samples of infants, were tested. The results were compared to that obtained by ferric reducing antioxidant power" (FRAP) as a standard method.

**Materials and Methods:**

Di-gentiobiosyl crocin was extracted and purified from Iranian saffron as previously described by us. Then, the CBA procedure using α-crocin was done in 2 different aquatic conditions, >50% or >90% of water. Results were analyzed by both the Bors method (calculating the relative rate constants= K_rel_) and the Tsimidou method (calculating the percent of -crocin bleaching inhibition=% Inh).

**Results:**

Our results indicated the following order of antioxidant potential for the above mentioned agents: ascorbic acid > uric acid > Trolox. However, these results are very similar to the data reported by others, but they are strongly related to the aqueous condition. In addition, uric acid showed different properties at different concentrations; so that it showed the antioxidant activity at low concentrations but it acted as a prooxidant at higher concentrations. Bilirubin interfered with this test, possibly because its maximum absorbance is close to the crocin. The obtained data for the antioxidant capacity of the serums was comparable with FRAP assay, except for the sample that contains high bilirubin concentration.

**Conclusion:**

In conclusion, it seems that CBA using the main fraction of crocin (-crocin) is a simple and useful method for determination of antioxidant potential of aqueous samples. In addition, the CBA ability to distinguish the samples that contain bilirubin or high uric acid content is helpful in clinical laboratories.

## Introduction

Biological free radicals are unstable molecules that contain unpaired electrons and can react with various organic substrates. In a normal cell, there is an appropriate prooxidant–antioxidant balance. However, this balance may be shifted toward the prooxidants when production of oxygen species is increased greatly, e.g. after ingestion of certain chemicals or drugs, or when the level of antioxidants are diminished. This is named the "oxidative stress" (1). Free radicals induce the damage of biomolecules and create toxic products that can alter gene expression and disrupt the physiological repair mechanisms (2).

The total antioxidant activity of plasma has been assayed by a number of different methods including: oxygen radical absorption capacity (ORAC) (3), determination of ferric reducing antioxidant power (FRAP assay) (4), Trolox equivalent antioxidant capacity (TEAC assay) (5), total radical trapping antioxidant parameter (TRAP assay) (6), and crocin bleaching assay (CBA) (7), which are extensively reviewed by Somogyi *et al* (1).

CBA was suggested by Bors *et al* in 1984 (8). It is an economical and sensitive method for measuring total antioxidant capacity of human plasma, natural compounds and plant extracts. In this assay, the extent of bleaching of crocin(s), the carotenoid from saffron, by peroxyl radicals is generated by thermal decomposition of azo-initiator (1, 8). In CBA, deletion of the hydrogen atoms and/or addition of a radical to the crocin(s) results in a disruption of the conjugated double bonds of its polyene backbone, thus bleaching of the solution (8). 

Crocin in the reduced form absorbs at 443 nm, but upon oxidation by radical species (R°), its absorbance reduces and finally disappears (2). This activity is expressed as the relative rate constant (K_rel_) or alternatively, as “percent of inhibition of crocin bleaching” (%Inh), which were suggested by Bors *et al *(8) and Ordoudi and Tsimidou, respectively (9).

According to the Bors method, relative rate constant (K_rel_) is derived from the following equation:

Vo/V=1+KAH/KC[AH]/[C] Eq.1

Where [AH] and [C] are concentrations of the mentioned antioxidant and crocin, respectively. K_AH_ and K_C_ are the rate constants of the reaction of radicals with AH and crocin, respectively. Second-order rate constants, V_0_ and V are obtained from kinetic curves in the absence or presence of antioxidant, respectively. These values are the slope of crocin absorbance plot (at 440 nm) against time, for the first 10 min of the reaction. Each experiment should be done at least five times, using five different concentrations of AH. Following determining the V_0_/V ratio at a known ratio of [AH] to [C], K_AH_/K_C _could be calculated by Eq.1 (7).

The second method was proposed by Tsimidou and her Ph.D. student, Ordoudi. We call it "Tsimidou method". According to this method, percentage of inhibition of crocin bleaching is derived from this equation: 

% Inh = [(ΔA_0_ -ΔA)/ΔA_0_)] 100 Eq.2

Where ΔA_0_ (absorbance of crocin alone) and ΔA (absorbance of the crocin solution after addition of AH) as in Eq. 1 can be obtained from only one [AH]/[C] ratio instead of at least five ratios required for the calculation of k_rel _or IC_50_ values (9).

CBA was considered to be applicable for “water-soluble” radical scavengers and related compounds. However, for "lipid-soluble" compounds, a modification of the assay was proposed using either canthaxanthin as a probe or a lipophilic initiator in organic solvent environment. R-Tocopherol and Trolox were commonly used as reference compounds under nonaqueous and aqueous conditions, respectively (9).

The proposed mechanism for the reactions chain in the CBA, are shown in the equations 3 to 7 (9), where the thermolabile azo-initiator is shown as RN; R and ROO are the radical and peroxyl radical species, respectively (9).

RN ^(heat)^ → 2R  + N_2 _ (Eq. 3)

R  + O_2_ ↔ ROO  (Eq. 4)

ROO  + crocin → ROOH + crocin  (Eq. 5)

ROO + AH → ROOH + A (Eq. 6)

A + crocin → AH + crocin (Eq. 7)

In the present study, we set up the CBA using Iranian saffron. Since in CBA, the active fraction of total crocin is α-crocin and its concentration in saffron obtained from different origin or different years is not the same, we used the -crocin that is purified in our Laboratory from saffron with the Ghaenate origin (Qayen, ). Then, some known antioxidants were chosen and were imposed to a competitive reaction with -crocin. The reduction of -crocin color is a good criterion to indicate the antioxidant potency of each compound. Validation of this technique to estimate the antioxidant activity of a few plasma samples was also examined. In addition, we tried to find the answer to this question: "Are the other antioxidants of saffron, e.g. crocetin, applicable for this assay?"

## Materials and Methods

Iranian saffron was obtained from Ghaenate (Qayen, Iran). L-ascorbic acid, uric acid, 2,2-diphenyl-1-picrylhydrazyl (DPPH), 2,2'-azino-bis(3-ethylbenzthiazoline-6-sulphonic acid) (ABTS) and 2,2'-azobis(2-aminopropane) dihydrochloride (AAPH, >98%) were purchased from Sigma-Aldrich Chem. Co.; BSA (bovine serum albumin) from Roche and glutathione and Trolox (6-hydroxy-2,5,7,8-tetramethylchroman-2- carboxylic acid) were purchased from Fluka. All materials were of analytical grade. 

Human plasma samples were collected from 8 infants and a young healthy, female blood donor.

Stock solution of AAPH (0.25 M) was daily prepared in 0.01 M PBS and stored at 4 °C.

*Apparatus*

A UV spectrophotometer, Shimadzu model 3101, was used for all absorbance measurements. A water bath (Sabalan Azmaye Tehran) for 10 min pre-heating of AAPH was applied. Other instruments were: centrifuge (Hettich N (UNIVERSAL 320 R)), fraction collector (Pharmacia LKB), shaker incubator (Heidolph Unimax 1010/Incubator 1000) and Freeze drier (FD-1 EYELA).

*Extraction and purification of -crocin*

Saffron extract was prepared and different fractions of components were purified according to the methods we explained previously (10-13). Two g of dried stigmas powder were mixed with 10 ml of n-hexane and was mixed for 10 min in shaker incubator, at room temperature in the dark. The extract was centrifuged at 4000 rpm for 10 min and the process was repeated three times. The pellet was finally extracted twice with 10 ml of 50% ethanol in water (v/v) and centrifuged at 4000 rpm for 5 min. The supernatant was applied on a glass column (2×80 cm) that was packed with neutral aluminium oxide 90-active and was eluted with 500 ml of 50% ethanol, followed by 500 ml of 50% ethanol containing acetic acid (4:1 v/v). Fractions of 4 ml were collected and their absorbencies were monitored at 250 and 440 nm. Picrocrocin was the first fractions which were eluted and then total crocins was separated. Fractions which contained total crocin (crocins) were run on another column filled with Silicagel G-60 equilibrated with water:ethanol:acetic acid (10:3:2 v/v) and the sample was eluted with the same solution at 12 hr. Fractions with similar absorbencies- at 440 and 250 nm were collected using fraction collector and were powdered with freeze drier. Only fractions containing -crocin, which is the main fraction of total crocin (11, 12) were used for the following experiments. The purity of the components during the extraction process was checked by spectrophotometry and TLC. The purity of the products was confirmed by HPLC and determination of melting temperature. In our previous work, H1NMR and IR spectroscopy were used for characterization of the mentioned components (11).

*CBA kinetic study*

Peroxyl radical scavenging activity was evaluated according to the protocol of Tubaro et al (7) with some modifications. Estimation of -crocin concentration to ~10 μM was based on the extinction coefficient which was the slope of plot of -crocin concentration versus absorption (ε= 12003 M-1 cm-1).

We made 2 stocks of -crocin in methanol. A high concentration stock with dilution of 40-50 µl of it to 1 ml using PBS, made a solution with A440 value ~1.3, and an usual stock that dilution of 400-450 µl of it to 1 ml using PBS, made a solution with A440 value ~1.3. For plasma samples, we just used more concentrated solution because plasma proteins which where imposed to methanol were denatured.

In the experiment based on the Bors method, the same volume of -crocin working solution was transferred into a series of 1.5 ml vials and various concentrations of each antioxidant (0.05-0.5 ml of 0.5 mM stock solution). Thus, the final concentration of antioxidant in the vials was ranged from 0 to 50 μM. According to the Tsimidou method, only one concentration of each antioxidant (10 μM) was used.

The reaction started with the addition of 150 μl of preheated (10 min at 39-40 C) AAPH stock solution. The final volume should reach 1 ml by addition of PBS. After 1 min stirring, the solution was transferred into a 1.5 ml quartz cell. In the Bors method, records were taken in the time course mode for a period of 10 min at 440 nm. In the Tsimidou method, the spectrum was recorded at time zero and after 10 min, between 200-600 nm and the absorption at 440 nm was used for calculations (10).

In Bors method, reduction in the absorbance within 10 min of reaction, in the absence (V0) or in the presence of AH (V) was calculated, and the relative rates (V0/V) were plotted against the [AH]/[C] ratios. The slope of the linear regression represents the relative rate constants (krel = kAH/kC) (10). The alkyl-peroxyl radical scavenging activity of each AH is presented in terms of TEVBors and TEVTsimidou (Trolox equivalent obtained on the basis of Bors or Tsimidou methods, respectively). Trolox equivalent values can then be easily produced if % InhAH or Krel AH is divided by the respective % InhTrolox or KrelTrolox (10).

We compared the antioxidant activity of plasma samples with both CBA and FRAP methods (14). The data was reported as mean±standard deviation (SD). In addition, the antioxidant activity of crocetin and picrocrocin were compared to crocin according to the Tsimidou method.

## Results

Two methods have been designed to explain the CBA results, that we call Bors and Tsimidou methods. These methods were explained extensively in the introduction and were applied to investigate the efficiency of -Crocin for CBA.

**Figure 1.**
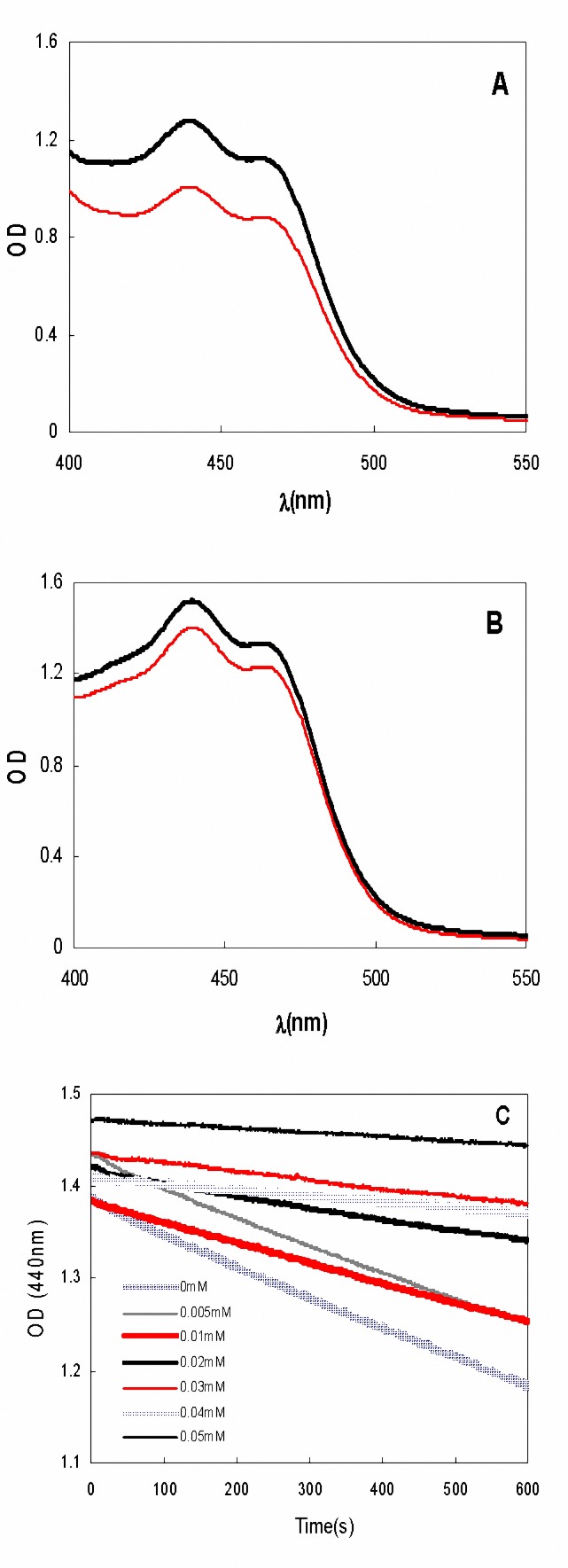
The spectra of  -crocin at beginning (black bold spectra) and after 10 min (red spectra) of the experiment, in the absence (A) or the presence of Trolox (B) as a second antioxidant.

In Tsimidou method the differences between the absorbance at 440 nm at the beginning of experiment (zero time) and after 10 min are the criteria for estimation of %Inh. Figures 1A & B show these plots for -crocin alone or in the presence of Trolox, respectively. In Bors method decrease in the slope of the plot of -crocin absorbance during the limited time course of experiment in the absence (V_0_) and presence (V) of antioxidant (AH) is the criteria for K_rel_ estimation. Figure 1C shows the plots of -crocin absorbance versus time in the presence of zero to 50 M of Trolox. 

**Figure 2.**
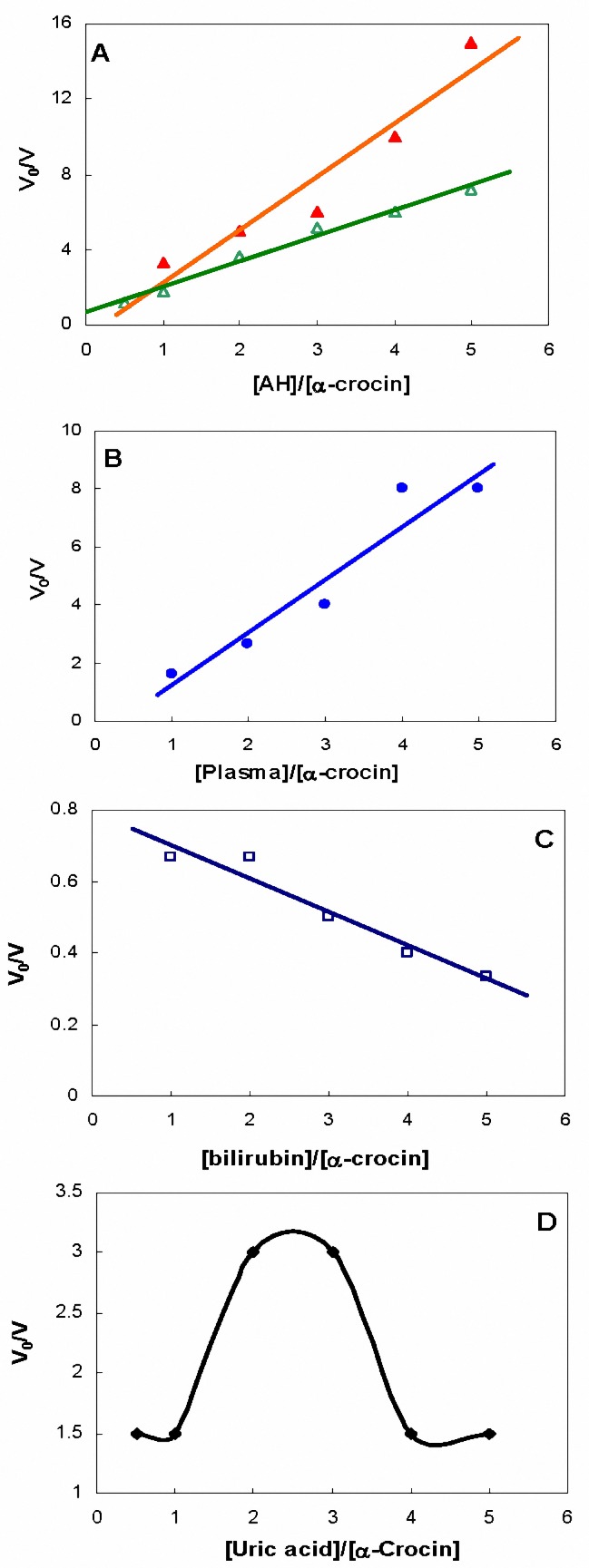
The plots of V_0_/V versus [AH]/[ -crocin] was determined by Bors method in the presence of (A) ascorbic acid (filled triangle, red) and Trolox (open triangle, green), (B) normal plasma sample, (C) bilirubin and (D) uric acid.

**Table1.** Trolox equivalent value obtained by both Bors (TEV_Bors_) and Tsimidou (TEV_Ts_) methods for the important antioxidants and adult plasma. These values for Trolox were equal to 1.

Antioxidant	Aqueous condition	TEV_(TS)_*	TEV_(B__ors)_*
DPPH	> 50% water	0.850±0.006	1.314±0.389
	> 90% water	0.320±0.002	0.209±0.096
Ascorbic acid	> 50% water	1.160±0.014	1.763±0.961
	> 90% water	1.577±0.014	6.764±0.325
Uric acid	> 50% water	0.730±0.075	0.847±0.099
	> 90% water	1.415±0.097	8.592±0.834
Plasma (Adult)	--------	0.891±0.035	1.419±0.240
BSA	--------	0.530±0.127	0.079±0.029
Glutathione	--------	0.343±0.106	0.040±0.032
Bilirubin	--------	-0.334±0.0007	-0.074±0.003

Each experiment was repeated at least three times and the data are presented as the mean±standard deviation.

**Table 2.** The antioxidant potential of plasma samples of infants (healthy orpatient) and normal adults, ascorbic acid and Trolox obtained by CBA in comparison with the FRAP.

Samples	TEV _(Ts)_*	
Plasma samples of infant with high bilirubin concentration	0.797±0.023	1512.000±73.539
Normal plasma sample of infant	0.464±0.038	453.000±39.598
Adult plasma	0.891±0.035	523.500±115.258
Trolox	1.0	556.000±91.924

Each experiment was repeated at least three times and the data are presented as the mean ±SD.

Figure 2A  shows the plot of V_0_/V versus [AH]/[-crocin] in the presence of ascorbic acid and Trolox. Figures 2B to 2D show the same parameters, as before, in the presence of normal plasma samples, bilirubin and uric acid, respectively. 

Table 1 shows the Trolox equivalent value determined based on both Bors (TEV_Bors_) and Tsimidou (TEV_Ts_) methods for 7 important antioxidants and adult plasma.

We also used several plasma samples of infants (healthy or patients) to estimate the antioxidant potential by CBA using -crocin in comparison with FRAP. In addition, the antioxidant activity of ascorbic acid and Trolox were compared using these methods. The results are shown in Table 2.

Addition of other saffron components such as crocetin to the solution had no significant effect on the results.

## Discussion

In all previous reports about crocin bleaching assay (CBA), total crocin, i.e. the mixture of various crocins with different number of glucose units have been used (7-9); however in the present study we used the pure crocin 1 [α-crocin or crocetin-di (β-D-gentiobiosyl) ester].

 The overall results of some papers about the order of antioxidant potential of some materials were the same, but the converse results was reported by others (7, 9,15). These differences may be depending on the type of crocin- that was used in the experiment. Total crocin is the mixture of various crocins, with different ratios. Thus, in the present study we used only -crocin to set up the crocin bleaching assay (CBA).

Results of the Trolox antioxidant potential obtained by both Tsimidou and Bors methods were similar (data not shown) and coincide with this fact that Trolox is a standard compound. As it is seen in Table 1, ascorbic acid at 50% of water and uric acid at 90% of water are the most powerful antioxidants. These data are coincident with those obtained in references 7 (by Tubaro *et al*) and 9 (by Ordoudi and Tsimidou), respectively. As CBA is introduced for aquatic assays, which is the biological condition, we propose to use >90% water in experiments and as a standard condition of this assay. Thus, the order of antioxidant potential of various materials were used in this study at %90 of water are similar, as determined by both methods of Tsimidou and Bors, and is similar to that obtained by others (1,9).

Antioxidant activities of ascorbic acid and uric acid were better in aquatic condition (>90% water) comparing to the solution containing less water (60:40 v/v of water: methanol), while DPPH showed better activity in the water/methanol solution. The results in Table 1 also indicated more activity of BSA and glutathione in the aquatic condition (this assay was just done in aquatic condition, to prevent the proteins denaturation caused by methanol). The importance of these results is due to the fact that methanol was known as a good solvent for DPPH (16), but these proteins are water soluble. Ascorbic acid is a flavonoid with different degrees of hydroxylation and its total number of hydroxyl groups influences its antioxidant activity (17). In the medium containing more than 90% water, OH groups have the best condition for optimum activity.

The antioxidant activity of uric acid is related to both water and uric acid concentrations. Howard and Haskins showed that solubility of uric acid increases in more aquatic medium (18) and after salvation its antioxidant activity is increased well. Determination of antioxidant activity of uric acid using the Bors method revealed the antioxidant and prooxidant activity at low and higher concentrations, respectively (Figure 2D). The results in Table 1  are related to its low concentration and are consistent with that reported by Nalsen (19).

Our results for determination of bilirubin antioxidant activity showed the reverse relation between this activity and bilirubin concentration. These results would be explained by the fact that both bilirubin and crocin maximum absorbance is about 440-450 nm. This explanation is confirmed by the negative values of bilirubin in Table 1. Similar results were reported by others (15). Thus, this method is not applicable for samples containing bilirubin higher than 3 mg/dl. Furthermore, crocetin addition showed no effect in the experimental solution that possibly is due to the similar maximum absorbance of crocetin and crocin. Thus, because of low solubility of crocetin in aquatic solutions, its replacement with crocin is not recommended.

Continuing our study, the validity of CBA using -crocin was tested for some plasma samples, as a preliminary work on a real sample. In the first row of Table 2, the antioxidant activity of plasma samples with high bilirubin concentration (3-20 mg/dl) was shown. It indicates the low antioxidant activity in CBA, but a high value FRAP. These results confirm that this method is not appropriate for samples containing high concentrations of bilirubin. The antioxidant activity of the other samples, with normal bilirubin content, showed the comparable values when they were determined by both methods.

## Conclusion

CBA using -crocin is a very useful method for determination of antioxidant activity of samples in the more aquatic conditions and in dilute solutions. Antioxidant activity of Trolox, as a standard compound, determined by both methods was consistent with each other. The Bors method needs more time, but its' results are very reliable. In contrast, the Tsimidou method is very rapid; and if it is repeated several times, its results are also reliable. In addition, our results indicated the validity of CBA using -crocin in the plasma samples with normal bilirubin concentration.
